# Comparison of tracheal temperature and core temperature measurement in living donor liver transplant recipients: a clinical comparative study

**DOI:** 10.1186/s12871-022-01853-9

**Published:** 2022-10-10

**Authors:** Seong-Mi Yang, Hye-Yeon Cho, Hee-Soo Kim

**Affiliations:** 1 Department of Anesthesiology and Pain Medicine, Seoul National University Hospital, 101 Daehak-ro, Jongno-gu, Seoul, 03080 Republic of Korea; 2 Department of Anesthesiology and Pain Medicine, Seoul National University College of Medicine, Seoul, Republic of Korea

**Keywords:** Body temperature, Living donor liver transplantation, Temperature monitoring

## Abstract

**Background:**

Body temperature is a vital sign, and temperature monitoring during liver transplantation is important. Tracheal temperature can be measured via an endotracheal tube with a temperature sensor on the cuff of the tube. This study aimed to investigate the accuracy and trending ability of tracheal temperature measurement compared to those of the core temperature measured at the esophagus and pulmonary artery (PA) in living donor liver transplant recipients.

**Methods:**

Twenty-two patients who underwent living donor liver transplantation (LDLT) were enrolled. Patients were intubated using an endotracheal tube with a temperature sensor placed on the inner surface of the tube cuff. Tracheal, esophageal, and PA temperatures were recorded at five time points corresponding to the different phases of liver transplantation. The tracheal and esophageal, tracheal and PA, and esophageal and PA temperatures were compared using Bland–Altman analysis, four-quadrant plot/concordance analysis, and polar plot analysis.

**Results:**

Bland–Altman analysis showed an overall mean bias (95% limits of agreement) between tracheal and esophageal temperatures of -0.10 °C (-0.37 °C to 0.18 °C), with a percentage error of 0.27%; between tracheal and PA temperatures, -0.05 °C (-0.91 °C to 0.20 °C), with a percentage error of -0.15%; and between esophageal and PA temperatures, 0.04 °C (-0.27 °C to 0.35 °C), with a percentage error of 0.12%. The concordance rates between tracheal and esophageal temperatures, tracheal and PA temperatures, and esophageal and PA temperatures were 96.2%, 96.2%, and 94.94%, respectively. The polar plot analysis showed a mean angular bias (radial limits of agreement) of 4° (26°), -3° (13°), and 2° (21°).

**Conclusions:**

Monitoring core temperature at the inner surface of the endotracheal tube cuff is accurate in all phases of LDLT with good trending ability; thus, it can be an excellent alternative for monitoring during LDLTs.

## Background

Body temperature is a vital sign monitored during surgery. Maintaining normothermia, a key responsibility of anesthesiologists, is important as temperature derangements can both cause and indicate disease [1]. Intraoperative hypothermia of < 35 °C frequently occurs during abdominal surgery and is associated with various postoperative complications [2–4]. Body temperature is monitored at various locations, such as the esophagus and nasopharynx [5]. In addition, the rectal and bladder temperatures reasonably estimate the core temperature. However, temperatures at the rectum and bladder have been reported to be lower than those at the esophagus during abdominal surgeries [6]. Therefore, body temperature is usually monitored using an esophageal temperature probe during general anesthesia.

Temperature monitoring during liver transplantation (LT) is important because thermoregulation is decreased in patients with end-stage liver disease. The use of a pulmonary artery catheter (PAC) is the gold standard for monitoring core temperature in patients with liver cirrhosis undergoing LT [7]. However, insertion of PAC is an invasive process that is not feasible for some liver transplant recipients. In such patients, the temperature is monitored in the esophagus, as in other abdominal surgeries. However, inserting the esophageal temperature probe requires caution as patients with cirrhosis have high-grade esophageal varices.

An endotracheal tube with a temperature sensor placed on the inner surface of the tube cuff was developed and introduced. This correlation between tracheal and core temperatures has been previously reported [6, 8, 9]. In cardiac patients, tracheal temperature accurately reflects the core temperature [6], and the temperature monitored on the surface of an endotracheal tube cuff in patients receiving therapeutic hypothermia after cardiac arrest accurately reflects the body temperature [9]. However, to the best of our knowledge, there are no reports on monitoring tracheal and core temperatures during LT.

Thus, this study aimed to investigate the accuracy and trending ability of tracheal temperature compared with those of the core temperature measured at the esophagus and pulmonary artery (PA) in liver transplant recipients. We hypothesized that tracheal temperature measured at the cuff of the endotracheal tube accurately reflects the core temperature measured at the esophagus and can replace esophageal probes in patients with end-stage liver disease undergoing LT.

## Methods

### Study design and patients

This clinical comparative study was approved by the Institutional Review Board of the Seoul National University Hospital (IRB No.2106–217-11,231) and was conducted in accordance with the tenets of the Declaration of Helsinki. Written informed consent was obtained from all the patients. This manuscript adheres to the applicable CONSORT guidelines. Patients older than 20 years and scheduled for living donor liver transplantation (LDLT) between August 2021 and February 2022 were evaluated. Patients who had a permanent catheter in the right internal jugular vein as well as contraindications for PA catheterization [10], such as presence of right-sided cardiac mass, tricuspid or pulmonic valve endocarditis, and severe tricuspid regurgitation, were excluded.

### Anesthesia and monitoring

**Fig. 1:**
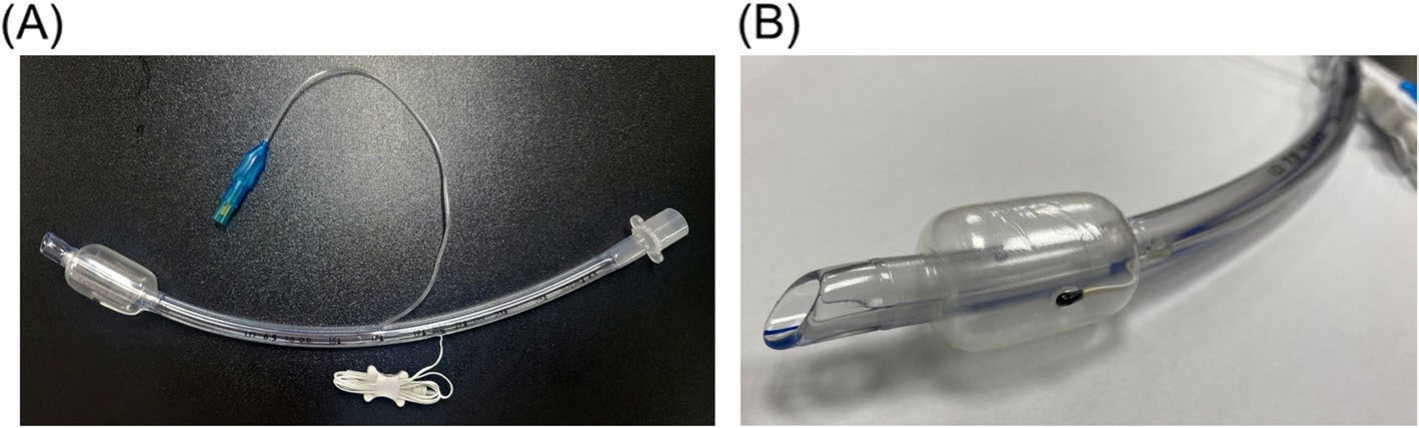
Endotracheal tube. **A** Endotracheal tube with a temperature sensor located on the inner surface of the cuff (Human Endo, Insung Medical, Korea). **B** Temperature sensor on the inner surface of the cuff of the endotracheal tube

The patient entered the operating room without any prewarming and premedication. Anesthesia was induced with propofol, remifentanil, and rocuronium and maintained with sevoflurane and remifentanil. Patient status index monitoring was performed with SedLine® (Masimo Inc., Irvine, CA, USA), targeting an index of 25–50. After the induction of anesthesia, endotracheal intubation was performed using an endotracheal tube with a temperature sensor located on the inner surface of the cuff (Human Endo, Insung Medical, Korea; Fig. 1A) to measure the tracheal temperature. The temperature sensor on the cuff is shown in more detail in Fig. 1B. Volume-controlled ventilation was performed at a tidal volume of 6–8 mL/kg. Anesthesia was maintained according to the liver transplant protocol at our hospital. An esophageal stethoscope was placed in the esophagus, and the esophageal temperature was monitored after the Levin tube was inserted. After central venous catheterization of the right internal jugular vein, a PAC (Swan-Ganz CCOmbo CCO/SvO2™; Edward Lifesciences LLC, Irvine, CA, USA) was inserted, and the temperature was monitored using the Vigilance II monitor (Edwards LifeSciences LLC, Irvine, CA, USA). The position of the PAC was confirmed using chest radiography. The Blanketrol® II (Cincinnati Sub-Zero Products, Cincinnati, OH, USA) was placed on the operating bed and the temperature was set at 37 °C throughout the whole operation. After anesthetic induction was completed, forced air warming using a Level 1 Snuggle Warm® Upper Body Blanket (Smiths Medical, Rockland, MA, USA) taped right above the nipple area and connected to a Level 1 Equator® warmer (Smiths Medical) set at 40 °C was used during the operation.

### Data collection

The temperatures within the trachea, esophagus, and PA were recorded at the following five time points corresponding to the different phases of LT during the surgery: (1) pre-anhepatic (1 h after anesthetic induction), (2) anhepatic 1 (recipient hepatectomy – inferior vena cava (IVC) clamping), (3) anhepatic 2 (IVC clamping – reperfusion), (4) reperfusion (5 min after reperfusion), and (5) neohepatic (1 h after reperfusion).

Demographic data, including the sex, age, body mass index, Model for End-stage Liver Disease score, Child–Pugh score, anesthesia time, operation time, estimated blood loss, and transfusion amount, were collected and analyzed.

### Primary and secondary endpoints

The primary endpoint was whether the tracheal temperature, measured via the endotracheal tube with a temperature sensor on the cuff, reflected the core temperature measured at the esophagus in liver transplant recipients. The accuracy and trending ability of tracheal to esophageal temperatures were compared at each phase of LT. The secondary endpoints were (1) the comparison between the tracheal and PA temperatures and between the esophageal and PA temperatures and (2) the correlation of the tracheal and esophageal temperatures with the gold standard PA temperature.

### Sample size considerations

In a previous study, the mean bias between the temperature measured at the tracheal mucosa and the temperature measured at the esophagus was -0.22 °C, and the standard deviation (SD) of mean bias was 0.11 °C [9]. Assuming a type I error of 0.05, a type II error of 0.1, and a maximum allowed difference of 0.5 °C, the minimum required number of pairs was 97, according to the calculation of the MedCalc software (version 15.2.2; MedCalc, Belgium). Considering a dropout rate of 10% due to technical difficulties, 108 pairs were required. Given that five measurements were recorded for each subject at the time points mentioned above, the minimum number of subjects required was 22.

### Statistical analysis

The patient characteristics are expressed as numbers (percentages), mean ± SD, or median with 25–75% interquartile range. The agreement between the tracheal and esophageal temperatures, tracheal and PA temperatures, and esophageal and PA temperatures was investigated using the Bland–Altman analysis with multiple measurements per subject [11, 12]. The mean bias and 95% limits of agreement (1.96 SD of the bias) were calculated. When the percentage error was within 10%, clinical compatibility was considered. Trending ability was analyzed using a four-quadrant plot analysis and polar plot analysis for each comparison. The trending ability was considered good if the concordance rate was > 92% in the four-quadrant analysis [13]. The concordance rate was calculated as the number of points in the upper-right and lower-left quadrants after excluding the exclusion zone (defined as 10%) divided by the total number of measures. Polar plot analysis shows agreement between the two methods by the angle from the line of identity (*y* = *x*) and the length of the vector [13]. The variables assessed from the polar plot analysis are the mean angular bias and radial limits of agreement. Trending ability was acceptable when the angular bias was less than ± 5°, and the radial limit of agreement was less than ± 30° [14]. All statistical analyses were performed using MedCalc software (version 15.2.2; MedCalc, Belgium), SigmaPlot 14.0, (Systat Software Inc, San Jose, CA) and R software (version 3.6.1. R Development Core Team, Vienna, Austria).

## Results

**Fig. 2:**
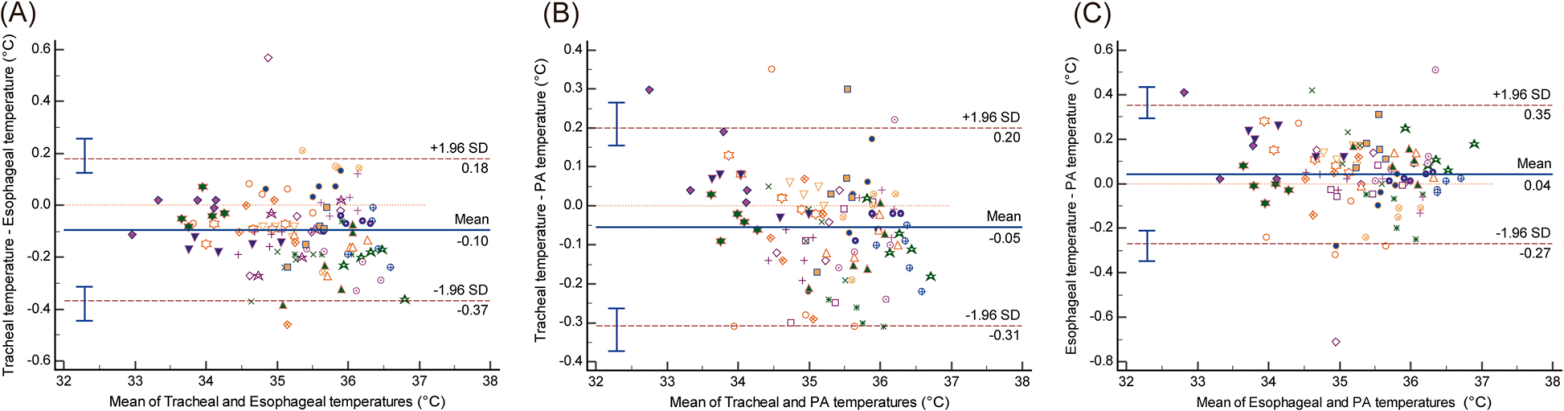
Bland–Altman plots. Bland–Altman plots for comparisons between the temperatures showing multiple measurements per subject. **A** tracheal and esophageal temperatures, **B** tracheal and pulmonary artery temperatures, and **C** esophageal and pulmonary artery temperatures

Twenty-two patients who underwent LDLT were enrolled. The clinicodemographic patient characteristics are shown in Table 1. The tracheal, esophageal, and PA temperatures from all 22 patients at all five points were recorded and used in the analysis, resulting in 110 time points with a total of 330 temperature measurements. Table 2 shows the mean bias, level of agreement, and percentage error between the tracheal and esophageal temperatures, tracheal and PA temperatures, and esophageal and PA temperatures. The overall mean bias (95% limits of agreement) between the tracheal and esophageal temperatures was -0.10 °C (-0.37 °C to 0.18 °C), and the overall percentage error was 0.27%. The overall mean bias (95% limits of agreement) between the tracheal and PA temperatures was -0.05 °C (-0.91 to 0.20 °C), and the overall percentage error was -0.15%. The overall mean bias (95% limits of agreement) between the esophageal and PA temperatures was 0.04 °C (-0.27 °C to 0.35 °C), and the overall percentage error was 0.12%. Figure 2 shows the Bland–Altman plots between the temperature measurements with multiple measurements per subject.

**Table 1:** Patient characteristics and perioperative variables

Variables	*n* = 22
**Baseline variables**	
Male	15 (68.2%)
Age, years	60 [55–66]
Height, cm	162.8 ± 9.4
Weight, kg	61.7 ± 11.3
Body mass index, kg/m^2^	23.0 ± 2.6
Patient comorbidities	
Hypertension, n	6 (27.3%)
Diabetes, n	6 (27.3%)
Preoperative medications	
Beta blocker, n	5 (22.7%)
Diuretics, n	7 (31.8%)
Insulin, n	1 (4.5%)
MELD score	9.3 [7.8–13.9]
Child–Pugh class A/B/C	13 (59.1%)/8 (36.4%)/1 (4.5%)
Etiology	
Viral-related liver cirrhosis	15 (68.2%)
Non-viral-related liver cirrhosis	6 (27.3%)
Others	1 (4.5%)
**Perioperative variables**	
Cold ischemic time, min	113.7 ± 37.8
Warm ischemic time, min	32.0 [26.0–36.0]
Anesthesia time, min	490.4 ± 89.6
Operation time, min	421.4 ± 89.2
Estimated blood loss, ml	2400 [1450–5300]
Crystalloid, ml	4325 [3600–5650]
20% albumin, ml	300 [200–550]
FFP, unit	0.0 [0.0–6.0]
RBC, unit	4.0 [0.0–10.0]
Pheresis, unit	0.0 [0.0–0.0]

Data are presented as n (%), mean ± SD, or median [interquartile range]

*Abbreviations: FFP* Fresh frozen plasma, *MELD* Model for End-Stage Liver Disease, *RBC* Red blood cell

**Table 2:** Differences in temperatures for living donor liver transplant recipients

	Tracheal vs. esophageal temperatures	Tracheal vs. pulmonary artery blood temperatures	Esophageal vs. pulmonary artery blood temperatures
Mean bias, °C	-0.10	-0.05	0.04
95% Confidence interval	-0.12 to -0.07	-0.08 to -0.03	0.01 to 0.07
Limits of agreement, °C	-0.37 to 0.18	-0.91 to 0.20	-0.27 to 0.35
Percentage error (%)	0.27	-0.15	0.12

**Fig. 3:**
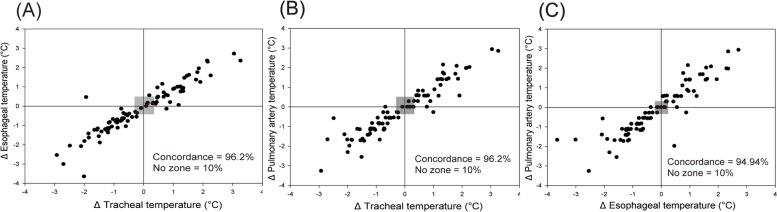
Four-quadrant plot analysis between the temperatures. An exclusion zone of 10% is shown in the gray zone. **A** tracheal and esophageal temperatures, **B** tracheal and pulmonary artery temperatures, and **C** esophageal and pulmonary artery temperatures

**Fig. 4:**
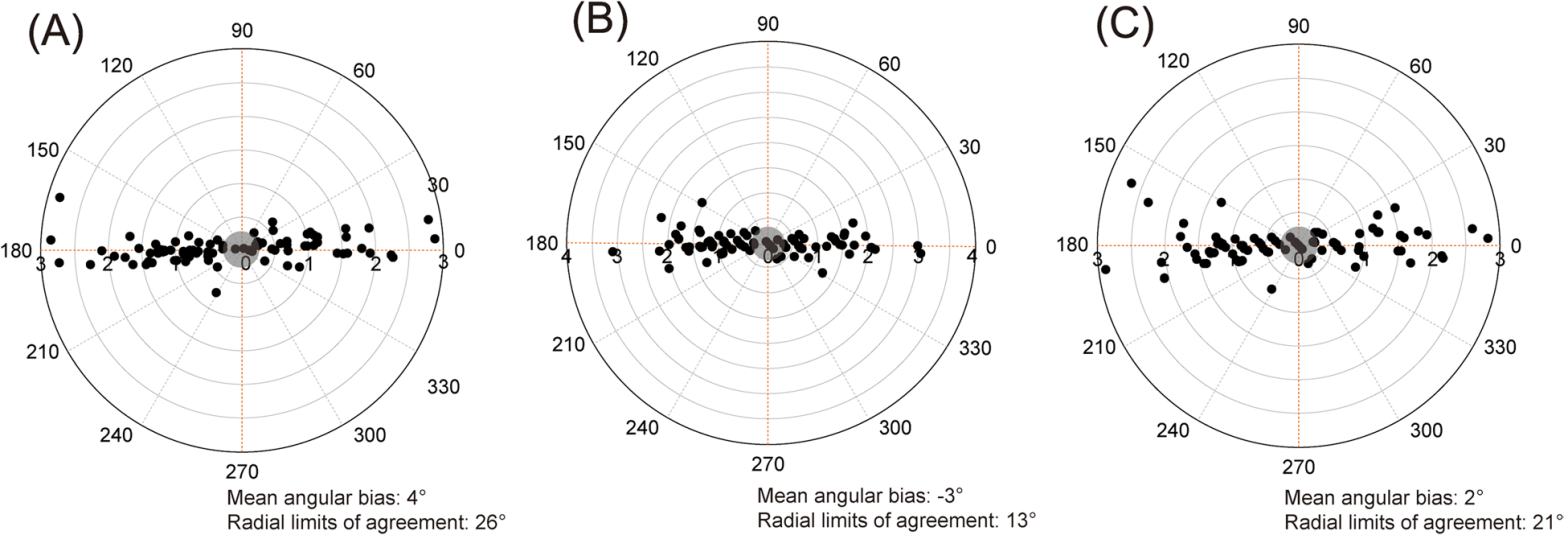
Polar plot showing the trending ability between the temperatures. An exclusion zone of 10% is shown in the gray zone. **A** tracheal and esophageal temperatures, **B** tracheal and pulmonary artery temperatures, and **C** esophageal and pulmonary artery temperatures

Figure 3 shows the four-quadrant plot analysis between the tracheal and esophageal temperatures, tracheal and PA temperatures, and esophageal and PA temperatures, with an exclusion zone of 10%. The concordance rates between the tracheal and esophageal temperatures, tracheal and PA temperatures, and esophageal and PA temperatures were 96.2%, 96.2%, and 94.94%, respectively. The trending ability using polar plot analysis is shown in Fig. 4. The mean angular bias between the tracheal and esophageal temperatures was 4°, and the radial limit of agreement was 26°. Compared with the tracheal and esophageal temperatures, the trending ability between the tracheal and PA temperatures was improved, with a mean angular bias of -3° and a radial limit of agreement of 13°. Lastly, the trending ability of the esophageal and PA temperatures showed a mean angular bias of 2° and a radial limit of agreement of 21°.

## Discussion

This study showed that tracheal temperature measured at the endotracheal tube's cuff correlated well with both the esophageal and PA temperatures at all phases of LDLT. The concordance rate and trending ability also showed high clinical acceptability. Monitoring temperature at the tracheal mucosa is accurate and can be used for monitoring core temperature in LDLT. To the best of our knowledge, this is the first report on tracheal temperature monitoring in liver transplant recipients.

Hypothermia can cause surgical wound infections [3], coagulopathy [4], and delayed postanesthetic recovery [15]. Normothermia is generally maintained using forced air blankets and fluid warming. Hypothermia in LT is more severe and frequent because of the following reasons: (1) underlying end-stage liver disease, (2) long duration of surgery, with the abdomen exposed for this long period, and (3) the liver graft entering the abdominal cavity is preserved in ice-cold saline, leading to a greater drop in temperature than that in other surgeries. Intraoperative hypothermia in LT is also associated with postoperative complications [16, 17].

The esophagus is the most obvious temperature monitoring site during general anesthesia and is well-perfused with blood from the core [1]. Tracheal temperature showed excellent accuracy and correlation with the temperature in the esophagus. A previous study on using an endotracheal tube containing a temperature sensor inside the cuff surface in treating patients with cardiac arrest showed good reliability [9]. The tracheal temperature probe on the balloon cuff of the endotracheal tube is usually placed just before the carina, which is close to the heart, enabling close monitoring of the core temperature. Using this endotracheal tube with an attached temperature probe eliminates the need to insert another temperature probe in patients undergoing general anesthesia.

The core body temperature, the average temperature of the core tissues, is best represented by the temperature of PA blood. Our findings show a good correlation between tracheal temperature and PA blood temperature, similar to esophageal and PA temperatures. It is possible to monitor the PA temperature using a PAC. However, as LDLT cases have increased, PAC insertion is not routinely performed in these cases. Monitoring PA pressure via PAC is still essential in patients with significant pulmonary hypertension [18]. However, it is unnecessary in patients without advanced cirrhosis and the signs of pressure overload in the right heart; therefore, many centers no longer use PAC as a clinical practice standard [19]. Furthermore, patients scheduled for incompatible ABO transplantations undergo plasmapheresis via a permanent catheter, which can be used as the large-bore catheter during surgery, eliminating the need to insert an additional large-bore catheter and insertion of PAC. Monitoring the tracheal temperature can be an alternative for these patients, removing the need for an additional esophageal temperature probe to be inserted.

Temperature monitoring during LT can be performed at the esophagus, nasopharynx, and bladder. However, esophageal probes should be inserted cautiously, especially in patients with high-grade varices since bleeding may result. Thus, the probe may not be inserted as deeply as necessary for core temperature monitoring. The temperature probe must be positioned at the distal esophagus at the point of maximal heart sounds or more distally to avoid cooling by respiratory gases [1]. In addition, the reading is sometimes not accurate during the manipulation of the Levin tube, which is routinely placed to deflate and suction the stomach. The use of transesophageal echocardiography in LT is increasing [20] in many centers and the manipulation of the transesophageal echocardiography probe can also interfere with accurate measurements of the esophageal temperature probe. Monitoring the bladder temperature requires a certain amount of urine output [21–23], which cannot be guaranteed in LTs, especially in patients with hepatorenal syndrome. Furthermore, the nasopharyngeal temperature can be directly affected by respiratory tract airflow [16] and inserting nasopharyngeal probes can lead to major bleeding in patients with coagulopathy. Since intubation is performed using an endotracheal tube at the start of anesthetic induction, temperature monitoring can be started immediately when using an endotracheal tube with a temperature probe on the cuff’s inner surface, without any disturbance or additional insertion of catheters or probes.

Previous studies on temperature monitoring in the trachea have reported mixed results. An animal study in pigs showed that tracheal temperature is an accurate surrogate for monitoring mild hypothermia [24]. Compared with established standard sensors to monitor PA, tympanic cavity, and esophageal temperatures, a temperature sensor on the endotracheal tube cuff responded more quickly and accurately during *in vitro* and dog experiments [8]. A temperature sensor placed along the shaft of the endotracheal tube is less responsive to abrupt temperature changes than a sensor in the cuff region. The previous study also showed that using heated and humidified oxygen at large minute volumes had minimal effect on body temperature measurement [8]. Meanwhile, another study comparing tracheal temperature with deep esophageal temperature showed a poor correlation and concluded that tracheal temperature is insufficient for core temperature monitoring [25]. However, in this study, the tracheal monitoring probe was placed in the lumen of the endotracheal tube. In reports with high correlation and in our study, since the temperature probe was placed at the inner surface of the cuff of the endotracheal tube, the temperature measurements were not affected by ventilation.

This study has a few limitations. First, the sample size is relatively small. Our results in living donor liver transplant recipients, showing that the temperature at the tracheal mucosa can be monitored at the inner surface of the endotracheal tube's cuff, need to be verified in a larger study with patients with other underlying conditions and in other surgeries as well as in deceased donor liver transplantations. Second, no postoperative outcomes (e.g., postoperative sore throat) were evaluated. However, since the temperature probe was placed on the inner surface of the cuff, no additional complications were reported, and the complication rate was expected to be similar to that of standard endotracheal tubes. Third, ventilatory parameters were not analyzed. However, as mentioned above, because the position of the temperature probe did not interfere with the lumen of the endotracheal tube, no disturbance in ventilation was observed. Further high-quality research with a larger number of patients addressing the complications and ventilatory parameters with the use of this endotracheal tube is needed.

## Conclusions

The core temperatures measured at the trachea, esophagus, and PA were identical. Acceptable bias and percentage errors were found for measurements in these areas, indicating accuracy. The trending ability was also high. Thus, monitoring the temperature at the inner surface of the endotracheal tube's cuff is feasible in all phases of LDLT and can be an excellent alternative for temperature monitoring during LDLTs.

## Data Availability

The datasets used and analyzed during the current study are available from the corresponding author on reasonable request.

## Abbreviations

LT: Liver transplantation
PA: Pulmonary artery
LDLT: Living donor liver transplantation
IVC: Inferior vena cava
SD: Standard deviation
PAC: Pulmonary artery catheter
